# Improved methodology for efficient establishment of the myocardial ischemia-reperfusion model in pigs through the median thoracic incision

**DOI:** 10.7555/JBR.36.20220189

**Published:** 2023-01-20

**Authors:** Liuhua Zhou, Jiateng Sun, Tongtong Yang, Sibo Wang, Tiankai Shan, Lingfeng Gu, Jiawen Chen, Tianwen Wei, Di Zhao, Chong Du, Yulin Bao, Hao Wang, Xiaohu Lu, Haoliang Sun, Meng Lv, Di Yang, Liansheng Wang

**Affiliations:** 1 Department of Cardiology, the First Affiliated Hospital of Nanjing Medical University, Nanjing, Jiangsu 210029, China; 2 Department of Cardiac Surgery, the First Affiliated Hospital of Nanjing Medical University, Nanjing, Jiangsu 210029, China; 3 Medical Experimental Animal Center, Nanjing Medical University, Nanjing, Jiangsu 210029, China

**Keywords:** coronary artery ligation, myocardial ischemia-reperfusion injury, Bama pig, animal model

## Abstract

To investigate the feasibility and effectiveness of establishing porcine ischemia-reperfusion models by ligating the left anterior descending (LAD) coronary artery, we first randomly divided 16 male Bama pigs into a sham group and a model group. After anesthesia, we separated the arteries and veins. Subsequently, we rapidly located the LAD coronary artery at the beginning of its first diagonal branch through a mid-chest incision. Then, we loosened and released the ligation line after five minutes of pre-occlusion. Finally, we ligated the LAD coronary artery *in situ* two minutes later and loosened the ligature 60 min after ischemia. Compared with the sham group, electrocardiogram showed multiple continuous lead ST-segment elevations, and ultrasound cardiogram showed significantly lower ejection fraction and left ventricular fractional shortening at one hour and seven days post-operation in the model group. Twenty-four hours after the operation, cardiac troponin T and creatine kinase-MB isoenzyme levels significantly increased in the model group, compared with the sham group. Hematoxylin and eosin staining showed the presence of many inflammatory cells infiltrating the interstitium of the myocardium in the model group but not in the sham group. Masson staining revealed a significant increase in infarct size in the ischemia/reperfusion group. All eight pigs in the model group recovered with normal sinus heart rates, and the survival rate was 100%. In conclusion, the method can provide an accurate and stable large animal model for preclinical research on ischemia/reperfusion with a high success rate and homogeneity of the myocardial infarction area.

## Introduction

With the improvement in living standards and the aggravation of aging population, the incidence of cardiovascular diseases in China continues to increase, posing a serious economic and social burden^[1–2]^. Myocardial infarction (MI) is one of the leading causes of cardiovascular morbidity and mortality worldwide, and ischemia/reperfusion (I/R) is considered a key risk factor for myocardial structure and function damage^[3–4]^. The epidemiological characteristics of MI have changed dramatically over the past decades, and the age-standardized MI incidence and mortality rates have increased in China. According to the "China Cardiovascular Health and Disease Report 2018", 11 million patients suffered from coronary heart diseases, and 4.5 million patients have experienced heart failure in China^[5–7]^. The total mortality rate of cardiovascular diseases still ranks first, higher than that of cancer and other diseases. MI not only seriously harms people's health and quality of life but also brings a huge burden and challenge to national finance and social insurance. This urgently requires the implementation of cost-effective policies and interventions^[8–9]^.

Establishing an MI and I/R model in preclinical research is essential for mechanistic research. Accurately evaluating cardiac function after I/R and the severity of myocardial injury is crucial for the choice of treatment scheme and prognosis of patients. Therefore, exploring the mechanisms and treatment strategies for MI has always been the focus in cardiovascular research^[10–12]^. Mice are the most widely used model animal for cardiovascular diseases worldwide due to their simple model construction method, minor trauma, and high survival rate^[13–15]^. However, considering the differences in anatomy and physiology between mouse and human hearts, any result of basic science research needs to be validated in large animal models before being applied to the clinic. The heart anatomy and blood vessel distribution of Bama pig are similar to the human heart in terms of body mass ratio, especially the less collateral cross distribution. Studies also confirmed that the hemodynamics of pig hearts are suitable for preclinical studies of myocardial I/R injury. Therefore, we chose the pig as the model to establish the myocardial I/R model^[16–19]^.

The established methods of I/R in miniature pigs includes drug induction, electrical stimulation, and coronary artery occlusion. Despite great efforts made in electrical stimulation and drug induction, the success rate of the I/R model is still unsatisfactory. The surgical methods of porcine ischemia-reperfusion can be divided into "open-chest coronary artery ligation" and "closed-chest coronary artery occlusion"^[20–22]^. Currently, the commonly used interventional methods of closed-chest coronary artery occlusion include coronary artery embolization, such as sponges, intracoronary injection of gel, and balloon occlusion of the coronary artery. However, the survival rate of the I/R model is limited by intraoperative ventricular fibrillation and thrombosis^[23–25]^. Although the method of thoracotomy and coronary artery ligation is traumatic, the thoracotomy operation is simple and conducive to the establishment of the MI model in line with the human heart morphology and hemodynamics^[26–27]^.

Therefore, the current study optimized the whole modeling process based on thoracotomy and coronary artery ligation, aiming to provide a more efficient and stable construction strategy for the pig MI model.

## Materials and Methods

### Experimental animals

Sixteen adult male miniature Bama pigs, aged approximately 12 months (Certificate No. 202149888), weighing (25.5 ± 2.775) kg, were purchased from Jiangsu Yadong Experimental Animal Research Institute. The pigs were raised in cages under a controlled temperature of 22 ℃ to 25 ℃ and kept on a 12-h light-dark cycle (feeding grade: conventional animal, breeder certificate No. 220213685). To perform the study, we first randomly divided 16 male Bama pigs into a sham group and a model group. The study was approved by the Ethical Committee for Experimental Animals of Nanjing Medical University (Approval No. IACUC-2005033).

### Surgical equipment and drugs

The surgical equipment used in the study included an echocardiography (IE33 digital ultrasonic scanner, PHILIPS Medical Systems, USA), a ventilator (Raywald R409PLUS, USA), a monitor (Raywald G3C, USA), an electrocardiogram (ECG, Nalong, RAGE-12L, China), and a defibrillator (Nalong, China). The surgical drugs used were listed in ***Table 1***.

**Table 1:** Surgical drugs

Drug name	Concentration	Company name	City	Province	Country
Epinephrine hydrochloride injection	1 mL: 1 mg	Beijing Yongkang Pharmaceutical Company Limited	Beijing	Beijing	China
Lidocaine hydrochloride injection	5 mL: 0.1 g	Hebei Tiancheng Pharmaceutical Company Limited	Cangzhou	Hebei	China
Benzylpenicillin potassium for injeection	0.5 g	Harbin Pharmaceutical Group Pharmaceutical General Factory	Harbin	Heilongjiang	China
Potassium chloride injection	10 mL: 1 g	China Otsuka Pharmaceutical Company Limited	Tianjin	Tianjin	China
Magnesium sulfate injection	10 mL: 2.5 g	Tianjin Jinyao Pharmaceutical Company Limited	Tianjin	Tianjin	China
Sodium chloride injection	10 mL: 0.09 g	China Otsuka Pharmaceutical Company Limited	Tianjin	Tianjin	China
Glucose injection	500 mL: 25 g	Beijing Fresenius Kabi Pharmaceutical Company Limited	Beijing	Beijing	China
Glucose injection	500 mL: 50 g	Beijing Fresenius Kabi Pharmaceutical Company Limited	Beijing	Beijing	China
Amiodarone hydrochloride injection	3 mL: 0.15 g	Sanofi-aventis (Hangzhou) Pharmaceutical Company Limited	Hangzhou	Zhejiang	China
Dopamine hydrochloride injection	2 mL: 20 mg	Shanghai Hefeng Pharmaceutical Company Limited	Shanghai	Shanghai	China
Heparin sodium injection	2 mL: 12 500 units	SPH NO.1 Biochemical & Pharmaceutical Company Limited	Shanghai	Shanghai	China

### Establishment process of the I/R model

#### Anesthesia of experimental animals

Induction of anesthesia: The pigs were fasted for 12 h and forbidden from drinking water for 4 h before the surgery. A pig was weighted, anesthetized with 50 mg/mL Zoletil 50 (Virbac, France) intramuscularly at 4–6 mg/kg, and then transported to the operating table. The pig was in the supine position, the limbs were fixed on the operating table, and the bilateral chest was fully exposed. Intravenous access was established by trocar in the ear margin vein, and 2.5% propofol [12 mg/(kg·h)] was injected intravenously to induce deep anesthesia.

Maintaining anesthesia: The ventilator parameters were adjusted to set a ventilation rate for Bama pigs at 20–30 times/min, respiratory ratio at 1:1.5–1:2.5, and tidal volume at 8–10 mL/kg initially. The endotracheal intubation and 2% isoflurane induction anesthesia were performed, with continuous intravenous injection of a polarized solution.

Recovery: No further anesthetic was added, but we waited for self-recovery after the basic vital signs were stable. Generally, the recovery time was about 10–30 min. If self-recovery was difficult, resuscitation drugs could be injected into the muscles behind the ears.

#### Preoperative preparation

After the pig was fixed in the supine position, the ear margin, chest, abdomen, and limbs were partially depilated with a leather knife. The electrodes were connected to continuously monitor and record the ECG changes (before, during, and after the operation). We disinfected, laid towels, prepared and checked surgical instruments. We adjusted the ventilator parameters as follows: a respiration rate of 25 times/min with a inhalation and exhalation ratio of 1:2; ventilation of 8–10 mL/kg body weight. Endotracheal intubation was performed, when the depth of anesthesia was sufficient.

#### Establishment of pig I/R model by median incision ligation

The pig was positioned supine and a median sternal skin incision was made with the upper edge 1 cm away from the sternal angle and the lower edge to the xiphoid process. The incision length was between 8 and 10 cm. The sternum was cut to the second intercostal space with an electric saw and cut to the right, preserving continuity of the manubrium sternum above the sternal angle. We opened the sternum with a chest opener, cut open the pericardium, and suspended it, when 100 U/kg of heparin was injected intravenously. After exposing the heart, we found the course of LAD, separated the arteries and veins, and passed through LAD with 5-0 sutures (LINGQIAO SUTURE, Ningbo Medical Needle Company Limited, Ningbo, Zhejiang, China) below the beginning of the first diagonal branch. The needle entry depth was 1 to 1.5 mm. After 5 min of pre-occlusion, the ligation wires were released, and the LAD was ligated 2 min later for 60 min. ECG and myocardial color changes were recorded before and after occlusion. Fatal arrhythmias and active bleeding were monitored during the operation. In the case of ventricular fibrillation, electric defibrillation combined with cardiac compression was given immediately, and a combination of cardiac surface and intravenous medication was administered simultaneously. After 60 min of ligation, the ligation line was loosened, the chest was closed, and the incision was sutured layer by layer. ECG, heart rate, respiration, blood pressure and oxygen saturation were continuously monitored during the operation. Emergency rescue measures were immediately taken, when abnormalities happened. The sham operation group underwent switching chests only.

#### Postoperative care

During anesthesia recovery, pigs were given a tidal volume of 8-10 mL/kg body weight. The corresponding indexes such as spontaneous breathing, pharyngeal reflex, heart rate, blood pressure, and pulse oxygen were continuously observed continuously after the operation. The endotracheal tube was removed after sputum suction, when all the above-mentioned indexes were normal. Infection was prevented by wrapping and fixing the wound with sterile gauze, and the dressing was changed regularly. Penicillin (4 million units, bid) was injected into the retroauricular muscle within three days postoperatively.

### Indicators of success of the pig I/R model

#### ECG detection

ECG was used to detect the ST-segment changes of pigs in the sham and model groups before, during, and after the operation.

#### Ultrasound cardiogram(UCG) detection

The changes in ejection fraction (EF) and fractional shortening (FS) were detected by UCG three days before, during, and seven days after the operation in the sham and model groups.

#### Myocardial markers detection

Venous blood samples were collected from the pigs in the sham and model groups 24 h before and after the operation. The myocardial markers, including cardiac troponin T (cTnT) and creatine kinase-MB isoenzyme (CK-MB), were detected using enzyme-linked immunosorbent assay (ELISA) kits (CK-MB ELISA kits, CB10105-Pg; cTnT ELISA kits, CB11635-Pg, COIBO BIO, Shanghai Coibo Biotechnology Company Limited, Shanghai, China).

#### Hematoxylin and eosin and Masson staining

At the 28 days after operation, the pigs in the sham and model groups were sacrificed, and the marginal area tissues of MI were obtained. The tissues were washed in PBS and fixed in 10% (v/v) formaldehyde, followed by dehydration in gradient ethanol and permeabilization in xylene. Then, the tissues were embedded in paraffin and cut into 4-μm slices by a Microslicer. Finally, the sections were stained with hematoxylin and eosin (H&E) and Masson staining kit (Sbjbio, Nanjing SenBeiJia Biological Technology Company Limited, Nanjing, Jiangsu, China) and observed by a light microscope (Philips 300, USA).

### Statistical analysis

Representative results were obtained through at least three independent experiments, and the data were presented as mean ± SD. The normality of the original data was tested using the Shapiro-Wilk test, and the results showed that the normality tests were significant (*P* > 0.05), indicating that the data were in line with normal distribution. Statistical analysis was performed with SPSS 19.0 software (IBM, USA), and images were acquired with GraphPad Prism 7 software (GraphPad Software, USA). The significance of the differences between the groups was compared using unpaired Student's *t*-tests. It was considered statistically significant when *P-*value was less than 0.05.

## Results

### Determination of successful establishment of the I/R model

**Figure 1:**
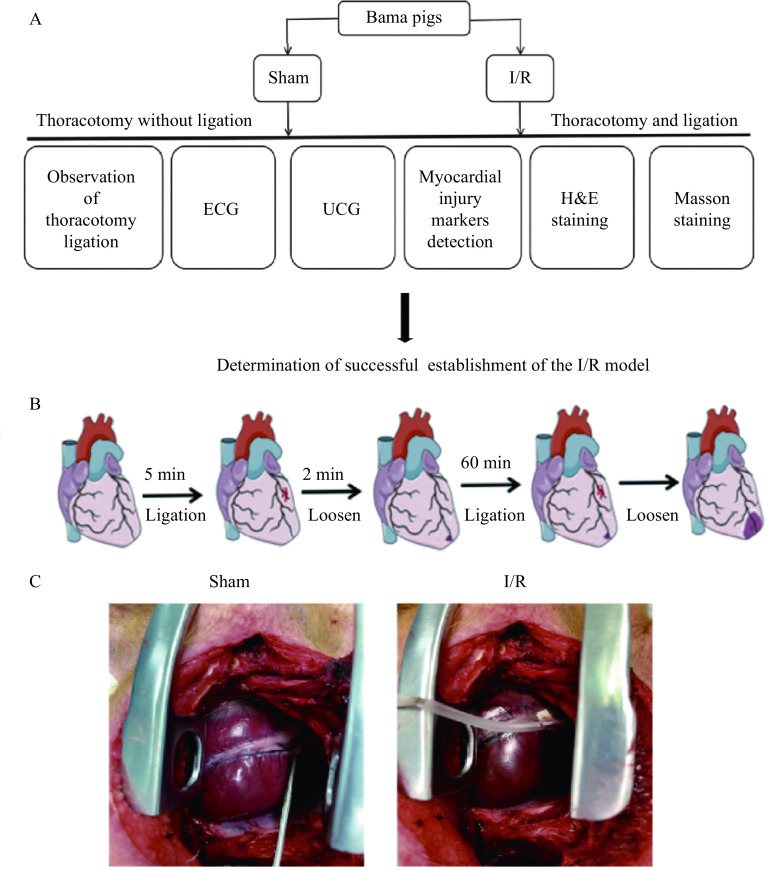
Construction of the Bama pig ischemia-reperfusion model.

Sixteen Bama pigs were used in this experiment, with eight in the sham operation group and eight in the model group. Compared with the sham group, the observed changes, which included ECG changes (ST-segment arching back up or lying down), UCG changes (abnormal ventricular myocardium movement, and the decrease of left ventricular EF and left ventricular FS, pathological changes (proliferation of fibrous tissues around the infarct area with inflammatory cell infiltration) and the elevated myocardial injury marker expression (troponin), in the model group suggested that the I/R model of eight pigs was successfully established. The success rate of modeling was 100% (***Fig. 1A*** and ***1B***).

### Observation of thoracotomy ligation

The form and cardiac impulse of pig myocardium in the sham group were natural without significant changes (***Fig. 1C***). The form and cardiac impulse of the myocardium in the model group were natural before ligation. After ligation at the beginning of the first diagonal branch of the LAD coronary artery, the blood supply area (anterior wall and apex of the left ventricle) supplied by the middle and far segment of the anterior descending coronary artery turned dark red. The motion of the ventricular wall weakened or disappeared (***Fig. 1C***).

### ECG detection

**Figure 2:**
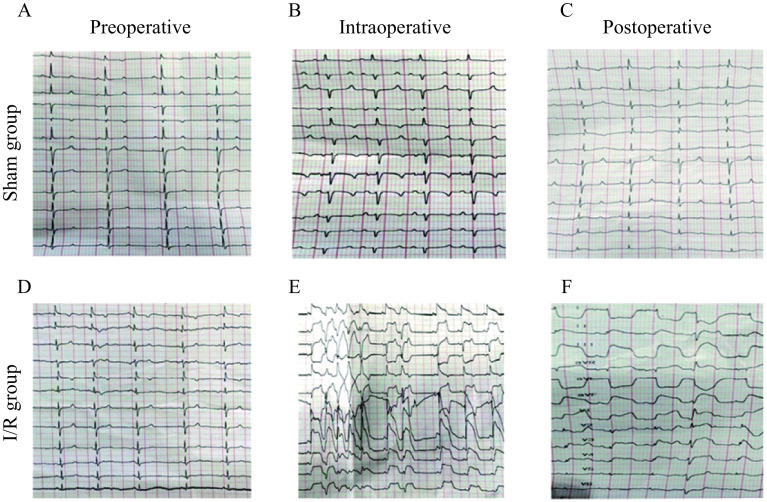
Changes of ECG in the model and sham groups before, during, and after operation.

The ECG of pigs in the sham group showed that the ST-segment position was at level with the baseline position before, during, and after the operation (***Fig. 2A–2C***). In the model group, however, the preoperative ECG leads (chest and limb leads) of pigs showed that the ST-segment position was at level with baseline position before I/R (***Fig. 2D***). The intraoperative ECG leads (chest lead and limb lead) showed that the P wave, QRS wave, and T wave all disappeared and were replaced with ventricular fibrillation waves with different sizes, shapes, and intervals (***Fig. 2E***). The postoperative ECG leads (chest and limb leads) showed that ST-segment elevation reappeared in multiple consecutive leads in the ECG, among which the elevation of V1-V3 leads was ≥0.3 mV and other leads were ≥0.1 mV. The new ST-segment horizontal downward movement was observed in Ⅰ and avL leads (***Fig. 2F*** and ***Table 2*****)**.

### Ultrasound cardiogram detection

**Figure 3:**
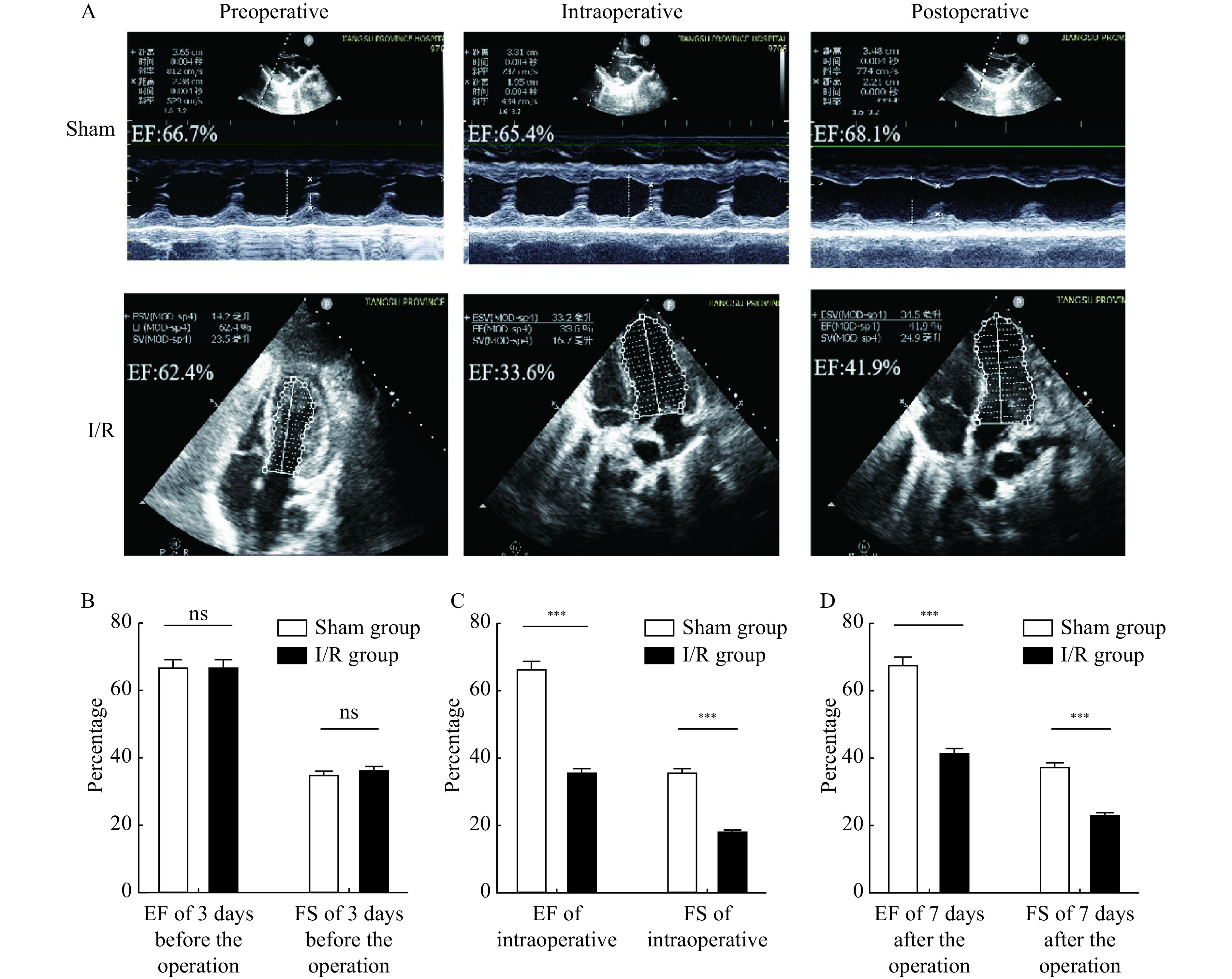
Changes of UCG in the model and sham groups 3 days before, 1 hour during, and 7 days after the operation.

Three days before the operation, there was no significant difference in EF and FS between the model and sham groups (EF: I/R, [67.7 ± 1.70]%, Sham, [67.1 ± 2.06]%; FS: I/R, [36.45 ± 1.08]%, Sham, [35.11 ± 1.41]%; all *P* > 0.05) (***Fig. 3A*** and ***3B***). During the intraoperative ischemia phase (one hour after ligation), the EF and FS in the model group were significantly lower than those in the sham group (EF: I/R, [35.9 ± 1.51]%, Sham, [66.9 ± 1.43]%; FS: I/R, [18.4 ± 1.16]%, Sham, [35.6 ± 1.06]%; all *P* < 0.001) (***Fig. 3A*** and ***3C***). UCG at seven days after the operation showed that the EF and FS in the model group were significantly decreased compared to those in the sham group (EF: I/R, [41.4 ± 1.84]%, Sham, [67.4 ± 2.07]%; FS: I/R, [23.11 ± 1.42]%, Sham, [37.41 ± 1.17]%; all *P* < 0.001) (***Fig. 3A*** and ***3D***).

### Myocardial injury marker detection

**Figure 4:**
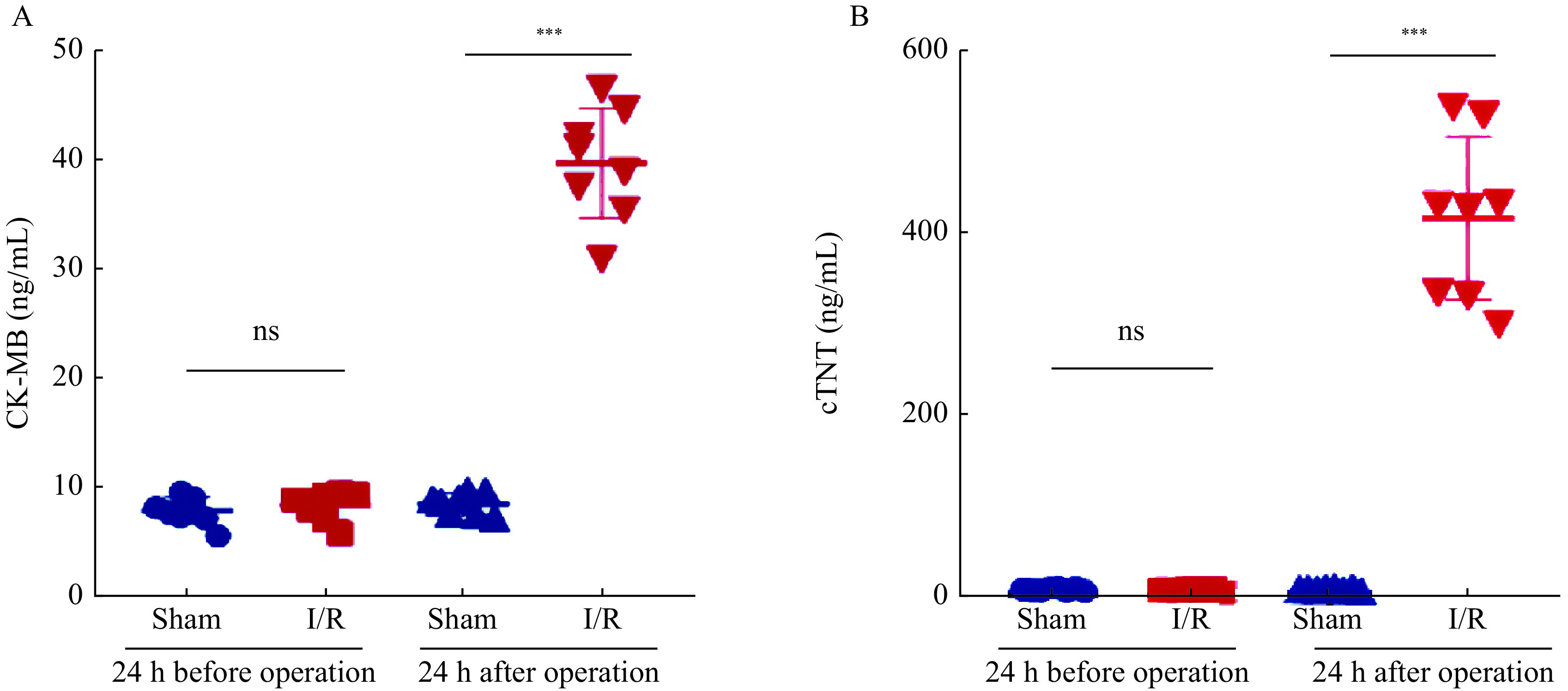
Plasma levels of myocardial injury markers in the sham and model groups.

The levels of cTnT and CK-MB in the model and sham groups were consistent at baseline (cTNT: I/R, [5.75 ± 1.62] ng/L, Sham, [6.08 ± 1.39] ng/L; CK-MB: I/R, [8.09 ± 1.35] ng/mL, Sham, [7.61 ± 1.24] ng/mL; all *P* > 0.05) (***Fig. 4A*** and ***4B***). Twenty-four hours after the operation, the cTnT and CK-MB of myocardial markers in the model group were significantly higher than those in the sham group (cTNT: I/R, [415.30 ± 89.50 ng/L, Sham: [5.78 ± 1.58] ng/L; CK-MB: I/R, [39.64 ± 5.07] ng/mL, Sham, [8.21 ± 0.97] ng/mL; all *P* < 0.001] (***Fig. 4A*** and ***4B***).

### Pathological examination

**Figure 5:**
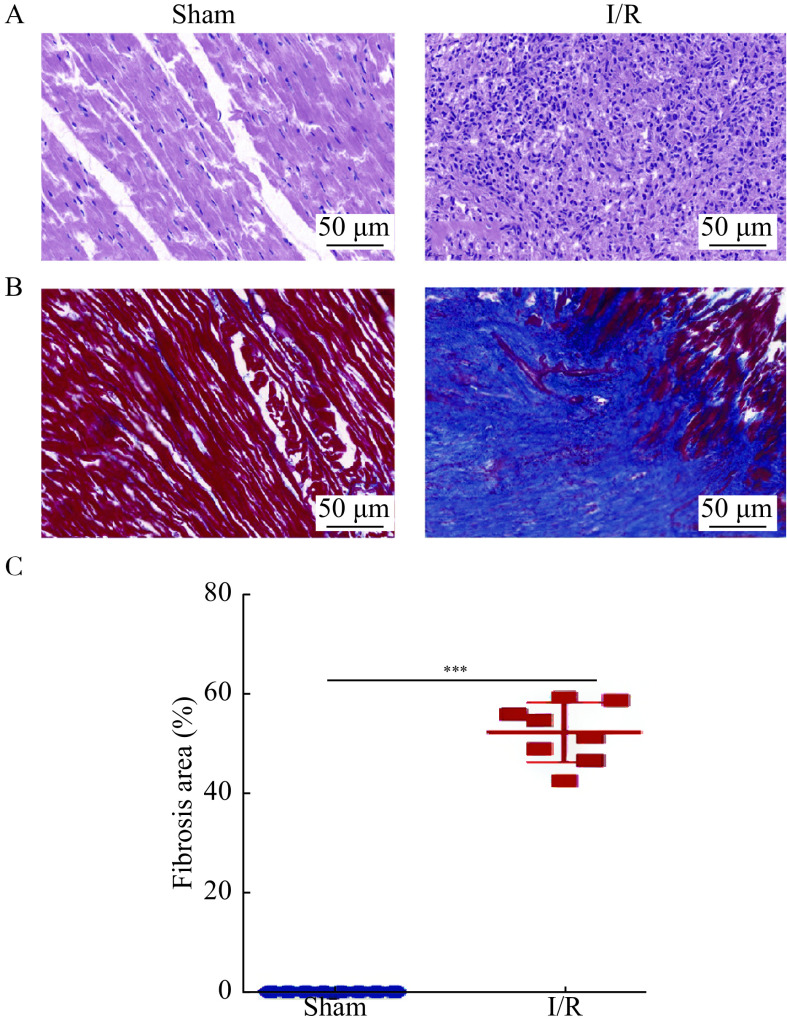
Pathological morphology of heart tissues in the sham and model groups.

The HE staining showed that MI was mainly located at the apex of the heart and the adjacent anterior wall of the left ventricle through the paraffin section of the model group, with inflammation infiltration (***Fig. 5A***). In addition, Masson staining showed a significant increase in fibrosis area in the model group, compared with the sham group ([52.21 ± 5.98]% *vs.* 0; *P* < 0.001) (***Fig. 5B*** and ***5C***).

### The intraoperative number of ventricular fibrillation and defibrillation in the model group

**Figure 6:**
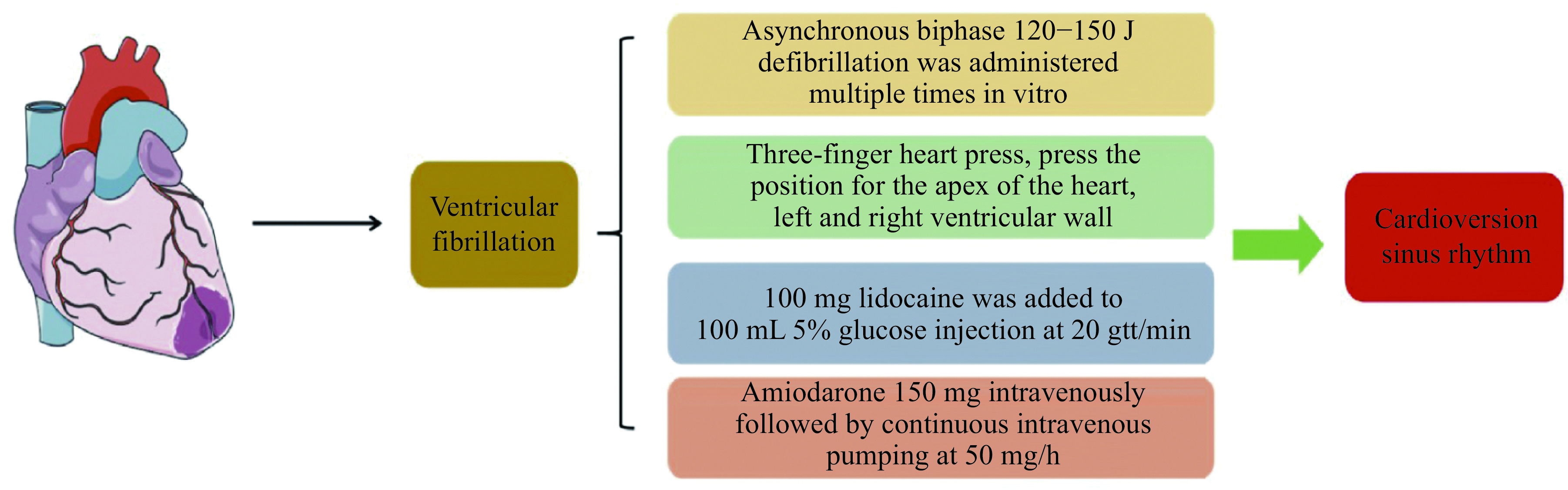
Emergency flow chart of treating ventricular fibrillation during ischemia-reperfusion operation.

Every pig in the I/R group experienced ventricular fibrillation. The specific incidence of ventricular fibrillation and defibrillation is shown in ***Table 3***. The typical ECG of pigs in the model group when ventricular fibrillation occurred is shown in ***Fig. 2E***. To treat ventricular fibrillation, we took the following emergency measures: (1) multiple external asynchronous biphasic 120–150 J defibrillation; (2) continuously pression with three fingers at the apex of the heart and the left and right ventricular walls in the defibrillation interval; (3) the sprinking of 1 mL adrenaline hydrochloride and lidocaine on the surface of the heart; (4) the administration of lidocaine 50 mg and the addition of 100 mg lidocaine into 100 mL 5% glucose injection at 20 gtt/min intravenously; (5) injection of amiodarone 150 mg intravenously, followed by continuous intravenous pumping at 50 mg/h. We repeated the procedure until the sinus rhythm was restored (***Fig. 6***).

**Table 3:** Frequency of ventricular fibrillation and defibrillation in pigs of the model group

No.	Heart rate before ligation (times/min)	Heart rate after ligation (times/min)	No. of ventricular fibrillation (times)	No. of defibrillation (times)
1	77	118	3	11
2	82	111	2	6
3	70	113	2	8
4	85	122	4	15
5	73	109	1	5
6	80	123	2	7
7	88	120	2	6
8	76	119	3	8

**Table 2:** Changes of ECG before and after the operation in the model group

No.	Heart rate before operation (times/min)	ST-segment position before operation	Heart rate after operation (times/min)	ST-segment elevation (mV)	ST-segment down (mV)
1	77	Level with baseline position	118	V1–V4 and Ⅲ raised upward with the bow of 0.1–0.6 mV	Ⅰ and aVF levels decreased by 0.3–0.6 mV
2	82	Level with baseline position	111	V1–V5 raised upward with the bow of 0.1–1.0 mV	Ⅰ level decreased by 0.6 mV
3	70	Level with baseline position	113	V1–V3 and aVR raised upward with the bow of 0.2–0.7 mV	Ⅰ and aVF levels decreased by 0.4–0.9 mV
4	85	Level with baseline position	122	V2–V4 and aVR are raised upward with the bow of 0.2–0.9 mV	aVF level decreased by 0.7–0.8 mV
5	73	Level with baseline position	109	V1–V2 raised upward with the bow of 0.3–0.8 mV	Ⅰ and aVF levels decreased by 0.5–1.0 mV
6	80	Level with baseline position	123	V3–V4 raised upward with the bow of 0.2–0.6 mV	Ⅰ level decreased by 0.7 mV
7	88	Level with baseline position	120	V4–V5 raised upward with the bow of 0.2–1.0 mV	Ⅰ and aVF levels decreased by 0.3–0.6 mV
8	76	Level with baseline position	119	V2–V5 raised upward with the bow of 0.2–0.7 mV	aVF level decreased by 0.5–0.6 mV
ECG: electrocardiogram.					

## Discussion

Balloon occlusion and thorax ligation are commonly used to construct the myocardial I/R model. Despite being characterized by minor trauma and convenience, balloon occlusion has an X-ray risk and a high experimental cost. On the other hand, it is difficult to achieve accurate and standardized occlusion of coronary vessels due to the lack of standard balloon positioning and high technical requirements^[28–29]^. To homogenize the construction of the pig I/R model, we adopted the median thoracic incision to construct the pig I/R model in the experiments.

Specific advantages of the median thoracic incision are as follows: (1) Due to the comprehensive control range of LAD and less collateral circulation in pigs, a high ligation position will enlarge the MI area. In contrast, a low ligation position may lead to an unsuccessful model. Therefore, the anatomical position of the pig heart can be fully exposed through the median thoracic incision so that the operator can accurately ligate at the beginning of the first diagonal branch of the LAD; (2) When intraoperative pigs have ventricular fibrillation, the operator can carry out timely rescue methods, such as electric defibrillation, three-finger press, and medication to improve the survival rate of pigs; (3) Before coronary ligation, the artery and vein should be separated first to reduce the occurrence of ventricular fibrillation during the operation and the mortality of pigs; and (4) The ligation of the median thoracic incision can fully expose the position of the heart so that the operator can closely detect the cardiac ultrasound value, thus providing comprehensive imaging evidence for the successful construction of the pig I/R model. Although the method had some advantages as described above, several disadvantages should be taken seriously: first, the wounds caused by the operation were relatively large; and second, the preoperative instrument preparation was more tedious.

To verify the successful construction of the model, the convinced indictors of I/R model, including the ECG, echocardiogram, myocardial injury index, and pathological staining, were compared between the sham and I/R groups. Compared with the sham group, we found that the ECG showed ST-segment elevations in multiple continuous leads in the model group. UCG results showed that EF and FS in the model group were significantly lower than those in the sham group. The difference was statistically significant at one hour and seven days after the operation. Twenty-four hours after the operation, the cTnT and CK-MB levels were significantly higher in the model group than those in the sham group. The HE staining results showed inflammatory cells infiltrating the myocardium of pigs in the model group. Masson staining showed a significant increase in collagen deposition in infarct size in the I/R group, compared with the sham group. The above results showed the successful construction of the I/R model in the model group.

To ensure the success of modeling and reduce the incidence of ventricular fibrillation, the following points should be paid attention to: (1) Fasting for eight to 12 h before the operation to ensure the anesthetic effect, and the dosage of anesthesia should strictly following the proportion of body weight. In addition, countermeasures should be prepared in advance, since anesthetics mostly have side effects. In this experiment, propofol was used to maintain anesthesia, and tracheal intubation was completed within 2 min after intravenous infusion to maintain ventilation to reduce the injury and death caused by hypoxia in the pigs. (2) Reasonable use of heparin (100 U/kg) before the operation and appropriate addition during the operation to prevent ventricular wall thrombosis. (3) Complete preoperative, intraoperative, and postoperative care, including maintaining a constant temperature in the feeding room, postoperative intramuscular injection of cephalosporin and penicillin, and adding drugs such as dacron and cephalosporin to the feed to prevent the occurrence of adverse results.

Compared with previously published modeling methods^[8]^, we adopted the median thoracic incision to establish the I/R model in the present study. The successful establishment of the pig I/R model was evidenced by the preoperative, intraoperative, and postoperative ECG, UCG, and pathological examinations. Moreover, a series of rescue measures were adopted to timely and effectively deal with the complications of porcine modeling (such as ventricular fibrillation), indicating that the strategy can steadily improve the success rate of the pig I/R model establishment. At the same time, the nursing and coping measures before, during, and after modeling were comprehensively optimized in experiments, which significantly improved the survival rate of the pigs after successful modeling, providing the reference and guiding significance for the construction of the myocardial injury model in preclinical research.

By enhancing surgical procedures and conducting various preoperative, intraoperative, and postoperative tests, we demonstrate that our method can provide an accurate and stable large animal model for preclinical research of I/R with a high success rate and homogeneity of the MI area.
